# Development of artificial intelligence in epicardial and pericoronary adipose tissue imaging: a systematic review

**DOI:** 10.1186/s41824-021-00107-0

**Published:** 2021-07-27

**Authors:** Lu Zhang, Jianqing Sun, Beibei Jiang, Lingyun Wang, Yaping Zhang, Xueqian Xie

**Affiliations:** 1 Radiology Department, Shanghai General Hospital, Shanghai Jiao Tong University School of Medicine, Haining Rd.100, Shanghai, 200080 China; 2 Shukun (Beijing) Technology Co., Ltd., Jinhui Bd, Qiyang Rd, Beijing, 100102 China

**Keywords:** Artificial intelligence, Epicardial adipose tissue, Pericoronary adipose tissue, Machine learning, Deep learning, Radiomics

## Abstract

**Background:**

Artificial intelligence (AI) technology has been increasingly developed and studied in cardiac imaging. This systematic review summarizes the latest progress of image segmentation, quantification, and the clinical application of AI in evaluating cardiac adipose tissue.

**Methods:**

We exhaustively searched PubMed and the Web of Science for publications prior to 30 April 2021. The search included eligible studies that used AI for image analysis of epicardial adipose tissue (EAT) or pericoronary adipose tissue (PCAT). The risk of bias and concerns regarding applicability were assessed with the Quality Assessment of Diagnostic Accuracy Studies-2 (QUADAS-2) tool.

**Results:**

Of the 140 initially identified citation records, 19 high-quality studies were eligible for this systematic review, including 15 (79%) on the image segmentation and quantification of EAT or PCAT and 4 (21%) on the clinical application of EAT or PCAT in cardiovascular diseases. All 19 included studies were rated as low risk of bias in terms of flow and timing, reference standards, and the index test and as having low concern of applicability in terms of reference standards and patient selection, but 16 (84%) studies did not conduct external validation.

**Conclusion:**

AI technology can provide accurate and quicker methods to segment and quantify EAT and PCAT images and shows potential value in the diagnosis and risk prediction of cardiovascular diseases. AI is expected to expand the value of cardiac adipose tissue imaging.

**Supplementary Information:**

The online version contains supplementary material available at 10.1186/s41824-021-00107-0.

## Background

Artificial intelligence (AI) refers to technology in which computers or other machines simulate human intelligence to enable problem solving (Chartrand et al. 2017). The progress of AI has gradually permeated the medical field, and its fusion with medical imaging is constantly deepening (Yu et al. 2018). A large amount of image data collected in clinical practice is a rich resource for AI, which significantly improves AI models. In radiology, AI excels at identifying complex image patterns and offers more quantitative and objective evaluation methods (Hosny et al. 2018b).

Machine learning and deep learning are AI methods. Machine learning refers to computer-based algorithms that perform tasks without explicitly programming and can conduct classification or regression tasks through learning patterns from engineered features (Chartrand et al. 2017). Radiomics is an emerging field widely used to mine predefined high-throughput features extracted from medical images to support clinical decision-making, and machine learning is the primary tool for feature selection and advanced model construction in radiomics (Lambin et al. 2012; Avanzo et al. 2020). Furthermore, the training of deep learning models obtains features and patterns directly from images rather than through engineered features (Hosny et al. 2018a). Deep learning is an end-to-end learning method that directly maps the entire learning process from the original data to the desired output (Chartrand et al. 2017).

Convolutional neural networks (CNNs) are one of the most popular deep learning architectures, which utilize convolution operations that effectively decrease the number of parameters, thus requiring less training data (Litjens et al. 2019). The application of CNNs in cardiac imaging has achieved remarkable results. For example, Betancur et al*.* exploited a CNN-based approach to identify obstructive coronary stenosis based on myocardial perfusion imaging (MPI) in single-photon emission computed tomography (SPECT), and the approach achieved an area under the curve (AUC) that was higher than that of MPI alone (per patient 0.80 *vs*. 0.78; per vessel: 0.76 *vs.* 0.73) (Betancur et al. 2018). With the growing interest in the investigation of epicardial adipose tissue (EAT) and pericoronary adipose tissue (PCAT), AI has begun to play an active and critical role in image analysis of EAT and PCAT.

EAT is located between the visceral pericardium and the myocardium and is in direct contact with the myocardium and coronary artery; EAT functions as an endocrine organ with metabolic and inflammatory activities (Gaborit et al. 2017). It can secrete bioactive molecules (adipocytokines, proinflammatory cytokines, growth factors, etc.) and affect the myocardium and coronary artery through vasocrine or paracrine (Iacobellis et al. 2005; Cherian et al. 2012). Studies have shown that EAT is associated with cardiovascular diseases (CVDs) such as arrhythmia, coronary artery disease, and heart failure (Topuz and Dogan 2017; Abe et al. 2018; Mancio et al. 2018). In addition, there is evidence that the PCAT around the coronary artery is the source of adipocytokines, which influences the development of coronary artery disease (Wang et al. 2013; Opincariu et al. 2020).

With the increasing potential of AI in disease assessment, the role of AI in the assessment of EAT and PCAT has attracted increasingly more attention. Since this field is novel but rapidly developing, we systematically review the latest progress of image segmentation, quantification, and the clinical application of AI in EAT and PCAT imaging.

## Methods

This study was conducted according to the preferred reporting items for systematic reviews and meta-analyses (PRISMA) 2020 statement (Page et al. 2021).

### Information sources and search strategy

We conducted a systematic literature search of PubMed and the Web of Science prior to 30 April 2021 using terms related to artificial intelligence and epicardial adipose tissue. The reference lists of relevant studies and reviews were checked to find other possible studies. The language was restricted to English. The detailed search strategies are listed in the supplement.

### Selection process and data collection

After removing duplicate articles, two reviewers (L.Z. and BB.J.) independently reviewed the titles, abstracts, and full texts to select eligible articles. Articles that did not use AI or that did not involve EAT or PCAT were excluded. The same two reviewers independently extracted the data from the included articles, including the article characteristics (authors, year of publication, and research purposes), sample sizes, imaging equipment, AI methods, and main results. In the process, the two reviewers resolved their disagreement via consensus with the third senior reviewer (XQ.X.).

### Study risk of bias assessment

Two reviewers (L.Z. and BB.J.) independently assessed the risk of bias and concerns regarding applicability of the included studies using the Quality Assessment of Diagnostic Accuracy Studies-2 (QUADAS-2) tool (Whiting et al. 2011) and resolved the disagreement by consultation with the third senior reviewer (XQ.X.). QUADAS-2 consists of four key domains: (1) patient selection, (2) index test, (3) reference standard, and (4) flow and timing. Considering the concerns regarding applicability in the index test domain, the studies with external validation were considered low concern.

## Results

### Study selection and study characteristics

**Fig. 1:**
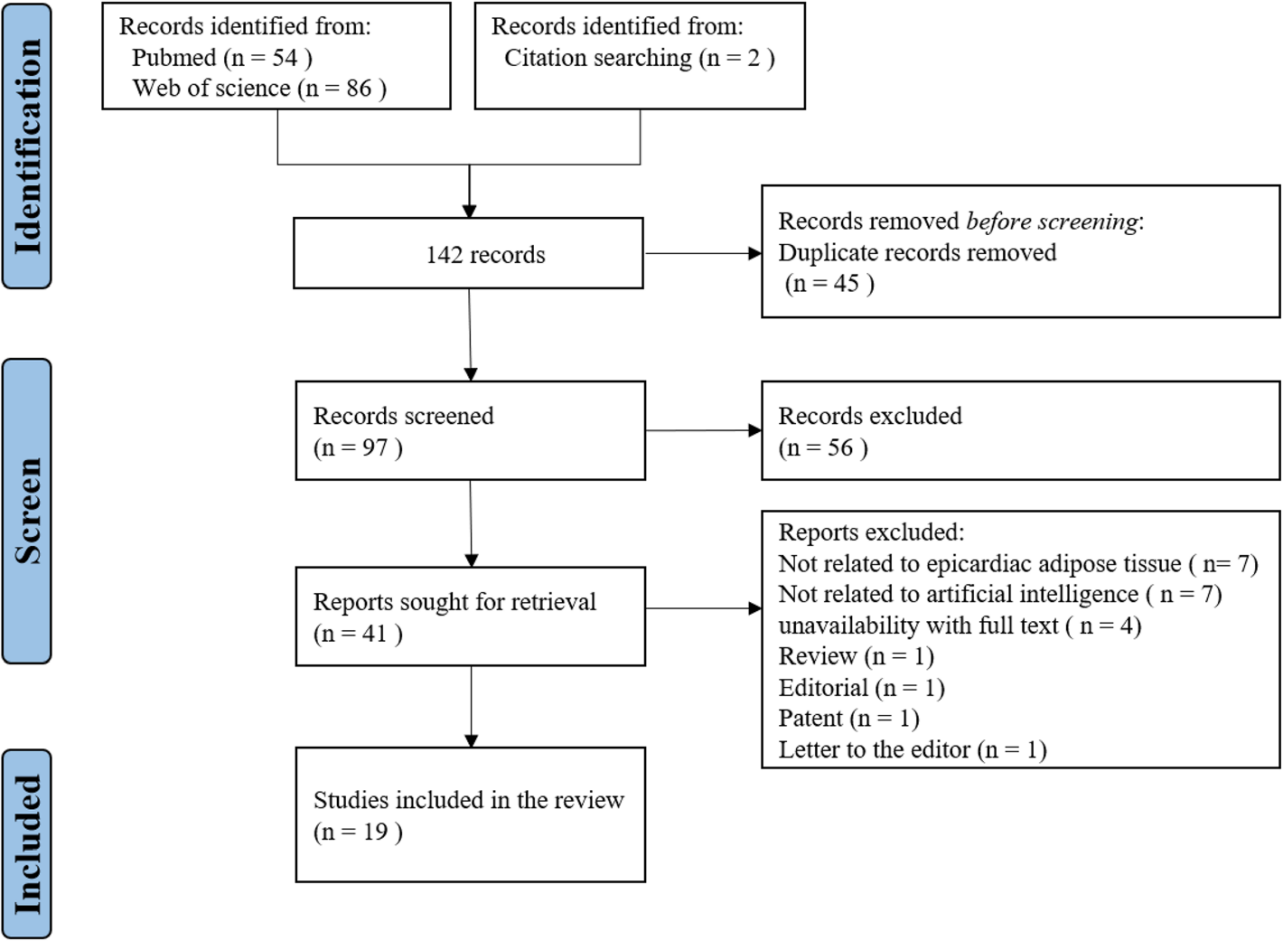
Flow diagram for screening and selection of articles

The initial search identified 140 citation records. Of these, 45 duplicate records were deleted, and 56 records were removed by browsing the titles and abstracts. After reading the full texts, 22 studies were excluded. Thereafter, 19 studies were finally included in the systematic review. Figure 1 depicts the flowchart of the selection process.

**Fig. 2:**
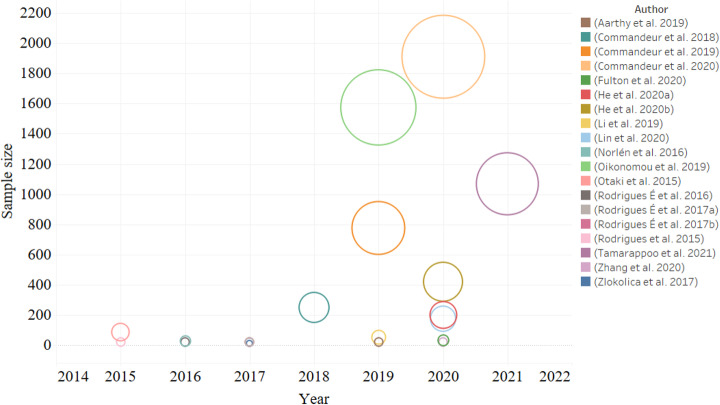
Sample size of the studies included in the systematic review by year of publication. The area of circle represents sample size

Figure 2 and Table 1 show the characteristics of the included studies. Of the 19 included studies, 15 (79%) used AI technology to segment and quantify EAT or PCAT, while 4 (21%) explored the clinical value of AI in the diagnosis and risk prediction of cardiovascular diseases. In terms of imaging equipment, 18 studies (95%) used computed tomography (CT), including 12 (63%) applying non-contrast scans and 6 (32%) performing coronary CT angiography (CCTA) scans. Only one study (5%) used cardiac magnetic resonance (CMR) imaging.

**Table 1:** Characteristics of studies included in the systematic review

Authors	Research purpose	Patients, *n*	Scan mode	AI method	Performance
(Rodrigues et al. 2015)	Automated segmentation of epicardial and mediastinal fats	20	Non-contrast CT	ML: Intersubject registration + RF	DSC = 0.968
(Rodrigues et al. 2016)	Automatic segmentation and quantification of cardiac fats	20	Non-contrast CT	ML: Atlas-based + RF	DSC = 0.977
(Rodrigues et al. 2017b)	Automated segmentation of epicardial fat	20	Non-contrast CT	ML: Genetic algorithms	The percentage of epicardial fat engulfed by the ellipse was 99.5%
(Norlén et al. 2016)	Automatic segmentation and quantification	30	CCTA	ML: Multi-atlas + RF + Markov random field	CC = 0.99 DSC = 0.91
(Zlokolica et al. 2017)	Semiautomatic EAT segmentation	10	CCTA	ML: Fuzzy c-means clustering + geometric ellipse fitting	DSC = 0.69
(Commandeur et al. 2018)	Segmentation and quantification of EAT	250	Non-contrast CT	DL: CNN	CC = 0.924 DSC = 0.823
(Commandeur et al. 2019)	Quantification of EAT	776	Non-contrast CT	DL: CNN	DSC = 0.871
(Li et al. 2019)	Automatic pericardium segmentation	53	Non-contrast CT	DL: U-Net	AUC = 0.87
(Aarthy et al. 2019)	Quantification of EAT	20	Non-contrast CT	DL: K mean clustering + CNN	CC = 0.803
(Fulton et al. 2020)	Segmentation of EAT	32	Cardiac magnetic resonance imaging	DL: Neural network	DSC = 0.56 ± 0.12
(Zhang et al. 2020)	Automatic epicardial fat segmentation and quantification	20	Non-contrast CT	DL: dual U-Nets + morphological processing layer	CC = 0.93 DSC = 0.91
(He et al. 2020a)	Automatic segmentation and quantification of EAT	200	CCTA	DL: 3D deep attention U-Net	DSC = 0.927
(He et al. 2020b)	Automatic quantification of myocardium and pericardial fat	422	CCTA	DL: Deep attention U-Net	ICC = 0.97 DSC = 0.88
(Otaki et al. 2015)	Prediction of impaired myocardial blood flow from clinical and imaging data (EFV)	85	Non-contrast CT	ML: Ensemble-boosting logitboost algorithms	AUC = 0.73 vs 0.67 (ML vs EFV)
(Rodrigues et al. 2017a)	Prediction of epicardial and mediastinal fat	20	Non-contrast CT	ML: Rotation forest + multi-layer perception regressor	Predicting mediastinal fat based on EAT: CC = 0.986 RAE = 14.4% Predicting EAT based on mediastinal fat: CC = 0.928 RAE = 32.5%
(Commandeur et al. 2020)	Predict the long-term risk of MI and cardiac death based on clinical risk, CAC, and EAT	1912	Non-contrast CT	ML: XGBoost	ML-AUC = 0.82 CAC-ACU = 0.77 ASCVD-AUC = 0.77
(Tamarappoo et al. 2021)	The long-term prediction of hard cardiac events	1069	Non-contrast CT	ML: XGBoost	ML-AUC = 0.81 CAC-AUC = 0.81 ASCVD-AUC = 0.74
(Oikonomou et al. 2019)	Radiotranscriptomic signature of perivascular fat improves cardiac risk prediction	1575	CCTA	ML: Radiomics-RF	For MACE discrimination: with radiomics signature-AUC = 0.88 without radiomics signature-AUC = 0.754
(Lin et al. 2020)	Radiomics analysis of PCAT to distinguish patients with MI	177	CCTA	ML: XGBoost	ML-AUC = 0.87 clinical features + PCAT attenuation-AUC = 0.77 clinical features alone-AUC = 0.76

*CCTA* Coronary computed tomography angiography, *ML* Machine learning, *DL* Deep learning, *RF* Random forest, *CNN* Convolutional neural network, *XGBoost* Extreme gradient boosting, *EAT* Epicardial adipose tissue, *PCAT* Pericoronary adipose tissue, *EFV* Epicardial fat volume (the volume of EAT), *MI* Myocardial infarction, *CC* Correlation coefficient, *DSC* Dice similarity coefficient, *AUC* Area under the ROC curve, *MSE* Mean square error, *RAE* Relative absolute error, *ASCVD* Atherosclerotic cardiovascular disease, *CAC* Coronary artery calcium, *MACE* Major adverse cardiovascular events

### Study quality assessment

**Fig. 3:**
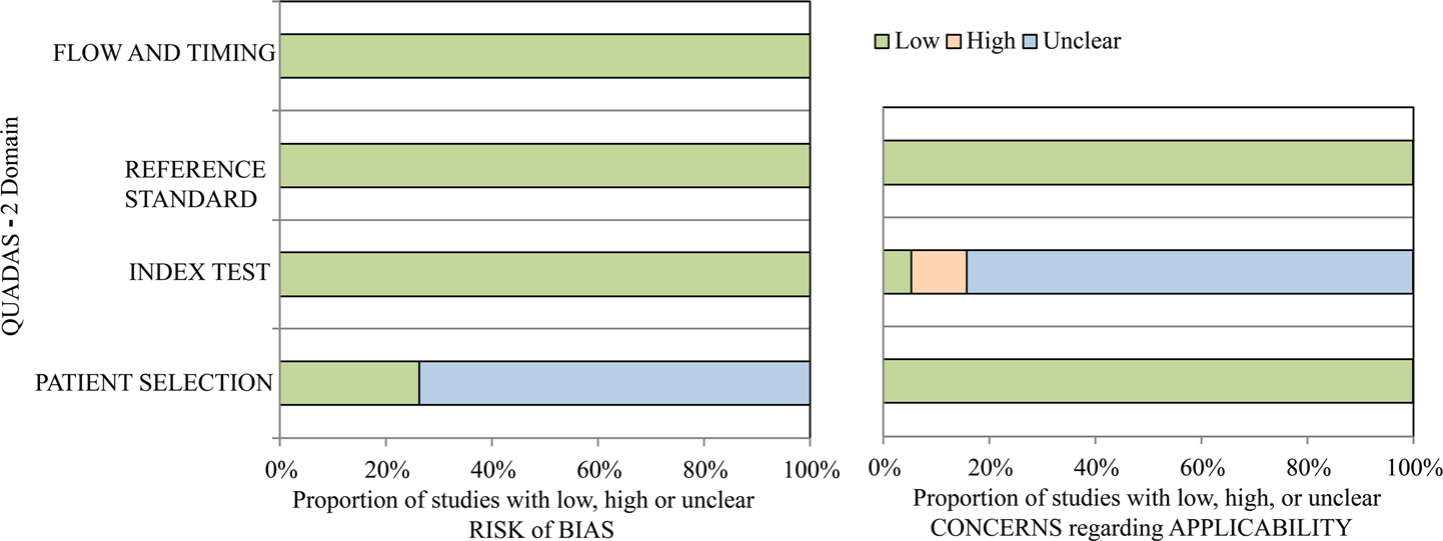
Assessment of individual risk of bias domains and concerns regarding applicability

Figure 3 and Table S1 represent the results of risk of bias and concerns regarding applicability according to the QUADAS-2 tool. For the assessment of bias risk, all 19 studies were rated as low risk in terms of flow and timing, reference standards, and index tests. In terms of patient selection, 5 studies (26%) were rated as low risk while 14 (74%) were rated as unclear risk. For the assessment of the concerns of applicability, all 19 studies were rated as low concern in terms of reference standards and patient selection. However, for the index test, only one study (5%) was rated as low concern because of the existence of external validation; 16 studies (84%) were considered unclear because they only conducted internal validation (cross or leave-one-out validation), and 2 studies (11%) were rated as high concern due to a lack of validation.

## Discussion

This systematic review summarized the research progress of AI in EAT and PCAT image analysis. An exhaustive literature search indicated that AI can improve the image segmentation and quantification of EAT or PCAT and can also be used as a tool for the diagnosis and risk prediction of coronary artery diseases based on EAT or PCAT. However, most of the studies have not applied external validation, which suggests that the generalizability of these studies remains to be improved.

### Segmentation of EAT and PCAT

Given the potential clinical value of EAT, the segmentation of EAT images is the crucial step for further quantitative analysis. In the traditional analysis mode, radiologists have to delineate the boundary of EAT manually. This procedure is user-dependent, time-consuming, and poorly reproducible, so it is necessary to develop an accurate, quicker, and reproducible method for EAT segmentation (Norlén et al. 2016; Commandeur et al. 2018). Researchers have developed machine learning algorithms to segment EAT. Rodrigues et al. utilized a genetic algorithm to optimize the parameters of an ellipse that was used to simulate the pericardium contour (Rodrigues et al. 2017b). After 10, 100, and 200 generations of genetic algorithm iteration, the percentage of epicardial fat engulfed by the ellipse was 97.3%, 98.8%, and 99.5%, respectively. Norlén et al*.* proposed a method to detect the pericardium using the random forest classification algorithm (Norlén et al. 2016). This method used feature-based multi-atlas regulations for spatial initiation in CCTA images. Finally, the segmentation was completed in global optimization through graph cuts, and the Dice similarity coefficient (DSC) between the proposed method and experts was 0.91. The average segmentation time was 51.9 s. The DSC is a commonly used measurement to evaluate the similarity between two structures, and its value ranges from 0 to 1. A higher DSC represents a higher similarity of image segmentation (Andrews and Hamarneh 2015).

Researchers have also explored the utilization of deep learning in EAT segmentation. Zhang et al*.* developed a dual U-Net CNN for the automatic segmentation and quantification of EAT (Zhang et al. 2020). Compared with a single U-Net (DSC = 0.766) and Seg-Net (DSC = 0.767), this method segmented the EAT of 20 patients with a mean DSC of 0.912.

In addition to tracking PCAT manually on axial images, PCAT can also be automatically segmented in 3D space. PCAT is considered adipose tissue whose radial distance from the outer wall of the coronary artery is equal to the mean diameter of the artery. A well-developed software package (Aquarius Workstation, TeraRecon GmbH) automatically segments PCAT radially outwards from the outer wall of coronary vessels in 3D space (Antonopoulos et al. 2017). PCAT is then defined by the CT value range (typically from − 190 HU to − 30 HU) of the adipose tissue within the segmented volume of interest.

In short, researchers have developed several AI-based methods for the automatic segmentation of EAT and PCAT. The studies applying AI technology to evaluate EAT (Hasebe et al. 2020) and PCAT (Oikonomou et al. 2019) showed good interobserver agreement, and the correlation coefficients were 0.93 and 0.94, respectively. These AI-based methods have the advantages of high accuracy, short processing time, and good reproducibility, which lay the foundation for further research.

### Quantification of EAT and PCAT

**Fig. 4:**
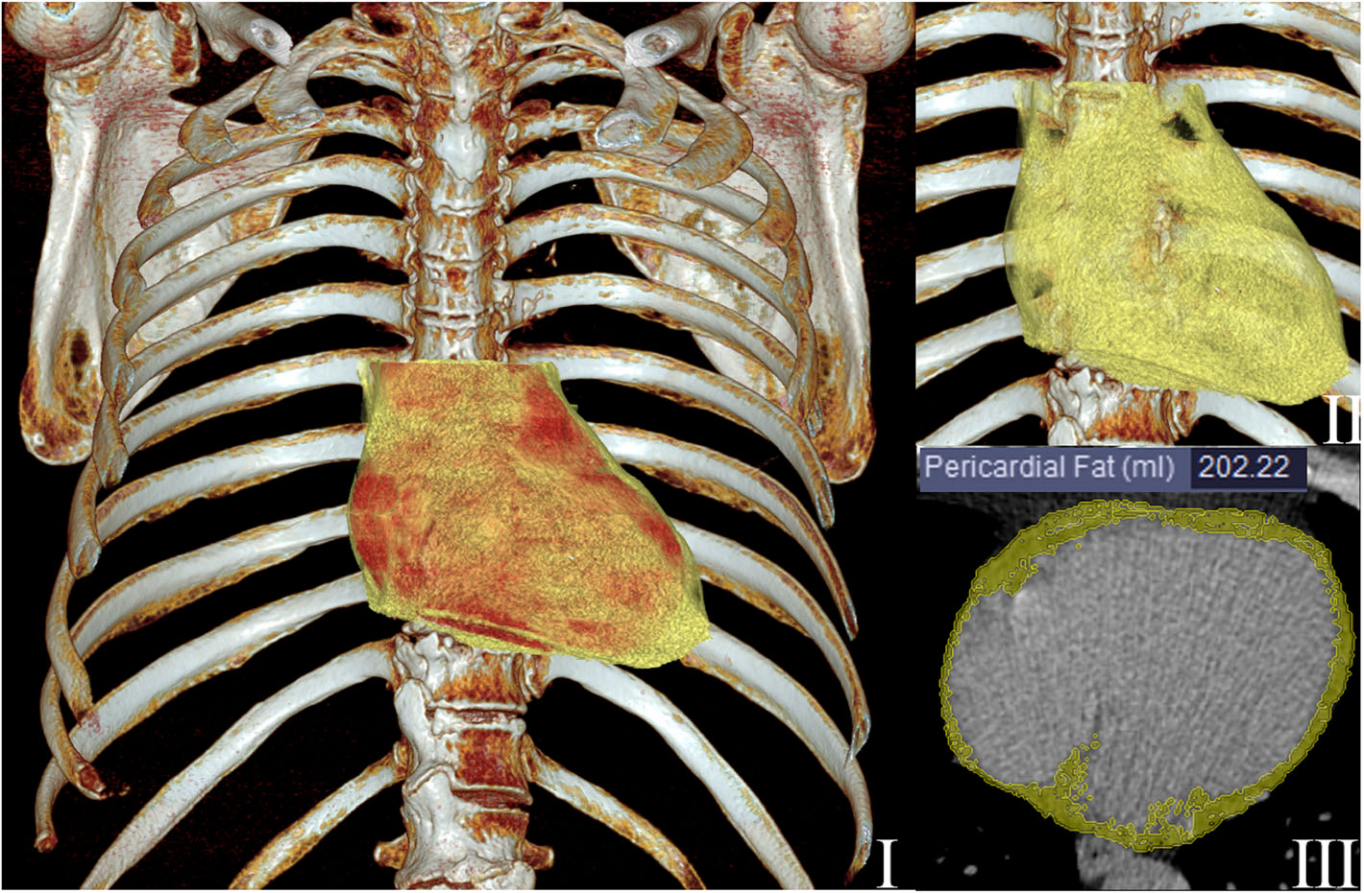
Schematic diagram of epicardial adipose tissue. Two 3D volume rendering images (**I** and **II**) and an axial CT image (**III**) show the heart (red color) and epicardial adipose tissue (yellow)

**Fig. 5:**
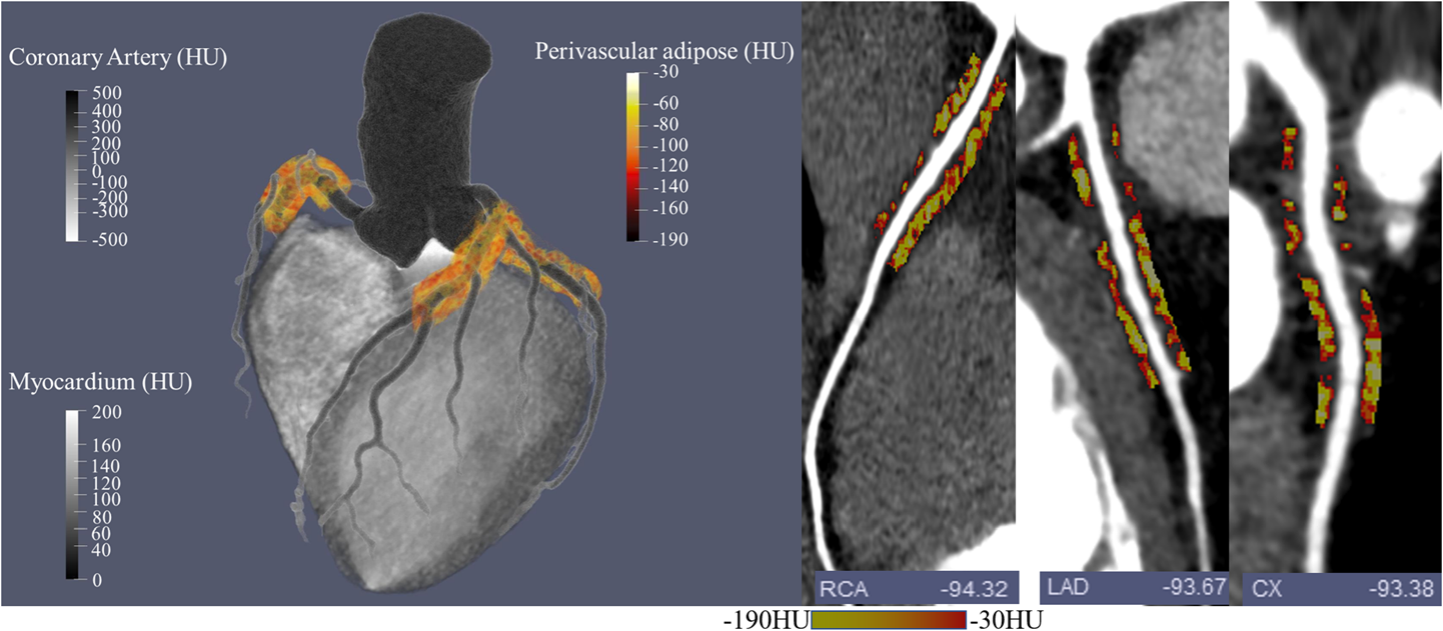
Schematic diagram of pericoronary adipose tissue and FAI. (left) 3D volume rendering image of pericoronary adipose tissue (orange color); (right) the numbers in the lower right corner represent the values of fat attenuation index. RCA = right coronary artery; LAD = left anterior descending artery; CX = circumflex artery; HU = Hounsfield unit

Quantification follows image segmentation. The quantitative parameters for EAT or PCAT are generally volume, area, and thickness. Commandeur et al*.* proposed a new multi-task framework using deep CNNs to quantify epicardial and thoracic adipose tissues (Commandeur et al. 2018). This method showed that the automatic volume quantification of epicardial and thoracic adipose tissue was strongly consistent with the manual method, and the median DSC was 0.823 and 0.905, respectively. The correlation coefficient between automatic and manual EAT volume quantification was 0.924. The automatic method for quantifying EAT took 25.63 ± 3.72 s per case, much shorter than the 10–11 min of the manual method. Figures 4 and 5 represent the image segmentation and quantification of EAT and PCAT, respectively.

### Clinical applications of machine learning in EAT

EAT plays an important role in the development of CVD through pathophysiological mechanisms (Mazurek et al. 2003; Cheng et al. 2008). Evidence suggests that abundant EAT is a predictor of adverse cardiovascular events (Mahabadi et al. 2013; Goeller et al. 2018). Since machine learning algorithms can maximize the data mining of image features and clinical factors, researchers have used them to analyze EAT. In a prospective study, Commandeur et al. developed a machine learning-derived risk score based on the extreme gradient boosting (XGBoost) algorithm, which combined clinical risk factors with the coronary artery calcium (CAC) score and automatically quantified EAT to predict the long-term risk of myocardial infarction (MI) and cardiac death (Commandeur et al. 2020). The machine learning-derived risk score (AUC = 0.82) performed better than the risk factors for atherosclerotic CVD (AUC = 0.77) and CAC score (AUC = 0.77). Otaki et al*.* applied the same algorithm (XGBoost) and found that the epicardial fat volume was an independent predictor of impaired myocardial flow reserve (Otaki et al. 2015). Compared with the CAC score (AUC = 0.66) and epicardial fat volume (AUC = 0.67), the AUC of the combined risk score integrating the CAC score and epicardial fat volume was 0.73, which improved the prediction ability of impaired myocardial flow reserve. In general, the existing evidence suggests that EAT, with the support of machine learning, can improve the diagnosis and prognostic prediction of CVD.

### Clinical applications of machine learning in PCAT

PCAT is the adipose tissue enveloping the coronary artery, which involves the evolution of atherosclerosis (Dey et al. 2010). The pericoronary fat attenuation index (FAI) is a novel imaging biomarker that maps the spatial changes in PCAT attenuation in CCTA images. The FAI has been used to identify subclinical coronary artery disease (Antonopoulos et al. 2017) and improve the risk prediction of all-cause cardiac mortality (Oikonomou et al. 2018). In addition to the FAI that simply represents the CT value, radiomics can mine the high-throughput quantitative information of the PCAT. Oikonomou et al*.* reported that the radiomics signature of the PCAT was associated with the fibrosis and vascularity of the PCAT, and the PCAT represented the adipose tissue remodelling caused by coronary inflammation (Oikonomou et al. 2019). In addition, they proposed a random forest method to analyze 1391 radiomic features of the PCAT extracted from 101 patients who had major adverse cardiac events (MACEs) within 5 years after undergoing CCTA and 101 matched controls. Their machine learning-based model performed better than the existing clinical risk factor model (Δ[C-statistic] = 0.126, *p* < 0.001) in predicting the outcome of MACEs. Lin et al. explored the ability of the radiomics signature of the PCAT in CCTA images to discriminate patients with MI from those with stable or no coronary artery disease (Lin et al. 2020). Using an XGBoost that combined clinical factors, PCAT attenuation, and radiomics features, their method significantly improved the discrimination ability of acute MI (AUC = 0.87) compared with a model with clinical factors and PCAT attenuation (AUC = 0.77) or clinical factors alone (AUC = 0.76). In brief, the implementation of machine learning can maximize the information extracted from PCAT and play an important role in the diagnosis and risk prediction of coronary artery disease.

### Multi-modal imaging of cardiac adipose tissue

Multiple types of equipment can be used to evaluate EAT, including CT, CMR, and echocardiography. CT offers volumetric visualization and quantification of cardiac adipose tissue with high spatial resolution. However, CT has the disadvantage of unavoidable radiation exposure. CMR is considered to be the best imaging method for adipose tissue because of its superb fat signal display (Wong et al. 2017). According to the results of Mahajan et al*.*, there was a significant correlation between CMR and autopsy for measuring the mass of adipose tissue with an intraclass correlation coefficient > 0.8 (Mahajan et al. 2013). The main disadvantages of CMR are its limited availability and high costs. Echocardiography is widely used, but the image quality is poor and not conducive to quantitative analysis. The quantification of EAT by echocardiography was limited to the thickness of adipose tissue on the right ventricular free wall (related to EAT thickness on CMR: R = 0.905) (Iacobellis et al. 2003; Gaborit et al. 2017).

Compared with the CT widely used in EAT analysis, research based on echocardiography and CMR is limited. In the included studies, only one used deep learning to segment EAT in CMR images with a low DSC of 0.56. More studies are expected to investigate EAT using AI and multi-modal imaging.

### Future prospects

The segmentation and quantification of EAT is an important basis of EAT research. Researchers have developed AI-based methods to make this process more accurate, quicker, and reproducible. In addition to developments in the experimental environment, more robust and generalizable AI methods are needed to assist the clinical application of EAT analysis.

As a booming field in recent years, radiomics enables the in-depth mining of image information. Based on EAT and PCAT, scholars have begun to use radiomics to diagnose and predict the risk of cardiovascular disease. However, to validate the clinical value of radiomics-based imaging biomarkers, more well-conducted multi-center studies are needed. Deep learning has become an auxiliary tool for image segmentation. On the basis of automatic segmentation of PCAT, an AI algorithm was used to quantitatively analyze and model the FAI, which expands the research of cardiac imaging. In addition to image segmentation, more AI-based research is expected to be launched to further explore the diagnostic and prognostic value of EAT and PCAT in CVD.

## Conclusion

With the recent development of AI, researchers have begun to apply AI to analyze EAT and PCAT images. AI-based methods provide accurate and quicker image segmentation and quantification. Although the publications are not numerous, the published studies have suggested the potential clinical application of EAT and PCAT in the diagnosis and risk prediction of CVD. Although AI research on cardiac adipose tissue is still in its infancy, the application of AI technology is expected to expand the clinical value of cardiac adipose tissue in cardiac imaging.

## Supplementary Information

**Additional file 1: Table S1.** Assessment of risk of bias and concerns regarding applicability according to the Quality Assessment of Diagnostic Accuracy Studies-2 (QUADAS-2) tool.

## Data Availability

Not applicable.

## Abbreviations

AI: Artificial intelligence
CNN: Convolutional neural network
AUC: Area under the curve
EAT: Epicardial adipose tissue
PCAT: Pericoronary adipose tissue
CVD: Cardiovascular disease
PRISMA: Preferred reporting items for systematic reviews and meta-analyses
QUADAS-2: Quality Assessment of Diagnostic Accuracy Studies-2
CT: Computed tomography
CCTA: Coronary computed tomography angiography
CMR: Cardiac magnetic resonance
XGBoost: Extreme gradient boosting
CAC: Coronary artery calcium
MI: Myocardial infarction
FAI: Fat attenuation index
MACE: Major adverse cardiac events
