# Supplementary material for: Development of artificial intelligence in epicardial and pericoronary adipose tissue imaging: a systematic review
Source: Eur J Hybrid Imaging. 2021 Jul 27;5:14. doi: 10.1186/s41824-021-00107-0 (PMC8313612; doi:10.1186/s41824-021-00107-0)
Supplement: Supplementary file 1 — Additional file 1: Table S1. Assessment of risk of bias and concerns regarding applicability according to the Quality Assessment of Diagnostic Accuracy Studies-2 (QUADAS-2) tool. [file 41824_2021_107_MOESM1_ESM.docx]

**Supplemental material**

**Search strategy**

**Pubmed**

(("artificial intelligence" or "machine learning" or "deep learning" or 'radiomics' or "convolutional neural network*" or "generative network" or "Generative Adversarial Network*") AND (“epicardial fat” or “pericardial fat” or “pericardial adipose tissue” or “paracardial adipose tissue” or “paracardial fat” or “epicardial adipose tissue” or “pericoronary adipose tissue” or “perivascular fat” or “perivascular adipose tissue” or “pericoronary fat” or “cardiac adipose tissue” or “cardiac fat” or “subepicardial adipose tissue” or “subepicardial fat”) AND (cardiac or cardio* or heart or coronary)) AND (English[Language])

**Web of Science**

TS = ("artificial intelligence" or "machine learning" or "deep learning" or 'radiomics' or "convolutional neural network*" or "generative network" or "Generative Adversarial Network*") AND TS = (“epicardial fat” or “pericardial fat” or “pericardial adipose tissue” or “paracardial adipose tissue” or “paracardial fat” or “epicardial adipose tissue” or “pericoronary adipose tissue” or “perivascular fat” or “perivascular adipose tissue” or “pericoronary fat” or “cardiac adipose tissue” or “cardiac fat” or “subepicardial adipose tissue” or “subepicardial fat”) AND TS = (cardiac or cardio* or heart or coronary)

Databases= WOS, BIOSIS, CSCD, DIIDW, INSPEC, KJD, MEDLINE, RSCI, SCIELO Timespan=All years

Search language=English

**Table S1** Assessment of risk of bias and concerns regarding applicability according to the Quality Assessment of Diagnostic Accuracy Studies-2 (QUADAS-2) tool

| **Study** | **Risk of bias** | | | | **Applicability concerns** | | | |
| --- | --- | --- | --- | --- | --- | --- | --- | --- |
|  | **Patient selection** | **Index test** | **Reference standard** | **Flow and timing** | | **Patient selection** | **Index test*** | **Reference standard** |
| Rodrigues et al. 2015 |  | ☺ | ☺ | ☺ | | ☺ |  | ☺ |
| Rodrigues É et al. 2016 |  | ☺ | ☺ | ☺ | | ☺ |  | ☺ |
| Rodrigues É et al. 2017b |  | ☺ | ☺ | ☺ | | ☺ | ☹ | ☺ |
| Norlén et al. 2016 |  | ☺ | ☺ | ☺ | | ☺ |  | ☺ |
| Zlokolica et al. 2017 |  | ☺ | ☺ | ☺ | | ☺ |  | ☺ |
| Commandeur et al. 2018 | ☺ | ☺ | ☺ | ☺ | | ☺ |  | ☺ |
| Commandeur et al. 2019 | ☺ | ☺ | ☺ | ☺ | | ☺ |  | ☺ |
| Li et al. 2019 |  | ☺ | ☺ | ☺ | | ☺ |  | ☺ |
| Aarthy et al. 2019 |  | ☺ | ☺ | ☺ | | ☺ | ☹ | ☺ |
| Fulton et al. 2020 |  | ☺ | ☺ | ☺ | | ☺ |  | ☺ |
| Zhang et al. 2020 |  | ☺ | ☺ | ☺ | | ☺ |  | ☺ |
| He et al. 2020a | ☺ | ☺ | ☺ | ☺ | | ☺ |  | ☺ |
| He et al. 2020b |  | ☺ | ☺ | ☺ | | ☺ |  | ☺ |
| Otaki et al. 2015 | ☺ | ☺ | ☺ | ☺ | | ☺ |  | ☺ |
| Rodrigues É et al. 2017a |  | ☺ | ☺ | ☺ | | ☺ |  | ☺ |
| Commandeur et al. 2020 | ☺ | ☺ | ☺ | ☺ | | ☺ |  | ☺ |
| Tamarappoo et al. 2021 |  | ☺ | ☺ | ☺ | | ☺ |  | ☺ |
| Oikonomou et al. 2019 |  | ☺ | ☺ | ☺ | | ☺ | ☺ | ☺ |
| Lin et al. 2020 |  | ☺ | ☺ | ☺ | | ☺ |  | ☺ |

☺Low Risk ☹High Risk Unclear Risk * The existence of external validation was considered low concern of applicability.

**Reference**

Aarthy D, Priya C, Sudha S (2019). Deep Learning for Quantification of Epicardial Fat from Non-Contrast CT. 2019 IEEE International Conference on Intelligent Techniques in Control, Optimization and Signal Processing (INCOS).

Commandeur F, Goeller M, Betancur J, Cadet S, Doris M, Chen X et al (2018) Deep learning for quantification of epicardial and thoracic adipose tissue from non-contrast CT. IEEE Trans Med Imaging **37**(8): 1835-1846

Commandeur F, Goeller M, Razipour A, Cadet S, Hell MM, Kwiecinski J et al (2019) Fully Automated CT Quantification of Epicardial Adipose Tissue by Deep Learning: A Multicenter Study. Radiol Artif Intell **1**(6): e190045

Commandeur F, Slomka PJ, Goeller M, Chen X, Cadet S, Razipour A et al (2020) Machine learning to predict the long-term risk of myocardial infarction and cardiac death based on clinical risk, coronary calcium, and epicardial adipose tissue: a prospective study. Cardiovasc Res **116**(14): 2216-2225

Fulton MR, Givan AH, Fernandez-del-Valle M, Klingensmith JD (2020) Segmentation of epicardial adipose tissue in cardiac MRI using deep learning. Proceedings of the SPIE - Progress in Biomedical Optics and Imaging **11317**: 113170Q (113177 pp.)-113170Q (113177 pp.)

He X, Guo BJ, Lei Y, Wang T, Curran WJ, Liu T et al (2020b) Automatic quantification of myocardium and pericardial fat from coronary computed tomography angiography: a multicenter study. Eur Radiol

He X, Guo BJ, Lei Y, Wang T, Fu Y, Curran WJ et al (2020a) Automatic segmentation and quantification of epicardial adipose tissue from coronary computed tomography angiography. Phys Med Biol **65**(9): 095012

Li Z, Zou L, Yang R (2019). A Neural Network-based Method for Automatic Pericardium Segmentation.

Lin A, Kolossváry M, Yuvaraj J, Cadet S, McElhinney PA, Jiang C et al (2020) Myocardial Infarction associates with a distinct pericoronary adipose tissue radiomic phenotype: a prospective case-control study. JACC Cardiovasc Imaging **13**(11): 2371-2383

Norlén A, Alvén J, Molnar D, Enqvist O, Norrlund RR, Brandberg J et al (2016) Automatic pericardium segmentation and quantification of epicardial fat from computed tomography angiography. J Med Imaging (Bellingham) **3**(3): 034003

Oikonomou EK, Williams MC, Kotanidis CP, Desai MY, Marwan M, Antonopoulos AS et al (2019) A novel machine learning-derived radiotranscriptomic signature of perivascular fat improves cardiac risk prediction using coronary CT angiography. Eur Heart J **40**(43): 3529-3543

Otaki Y, Hell M, Slomka PJ, Schuhbaeck A, Gransar H, Huber B et al (2015) Relationship of epicardial fat volume from noncontrast CT with impaired myocardial flow reserve by positron emission tomography. J Cardiovasc Comput Tomogr **9**(4): 303-309

Rodrigues É O, Morais FF, Morais NA, Conci LS, Neto LV, Conci A (2016) A novel approach for the automated segmentation and volume quantification of cardiac fats on computed tomography. Comput Methods Programs Biomed **123**: 109-128

Rodrigues É O, Pinheiro VHA, Liatsis P, Conci A (2017a) Machine learning in the prediction of cardiac epicardial and mediastinal fat volumes. Comput Biol Med **89**: 520-529

Rodrigues É O, Rodrigues LO, Oliveira LSN, Conci A, Liatsis P (2017b) Automated recognition of the pericardium contour on processed CT images using genetic algorithms. Comput Biol Med **87**: 38-45

Rodrigues ÉO, Conci A, Morais FFC, Pérez MG (2015). Towards the automated segmentation of epicardial and mediastinal fats: A multi-manufacturer approach using intersubject registration and random forest. 2015 IEEE International Conference on Industrial Technology (ICIT).

Tamarappoo BK, Lin A, Commandeur F, McElhinney PA, Cadet S, Goeller M et al (2021) Machine learning integration of circulating and imaging biomarkers for explainable patient-specific prediction of cardiac events: A prospective study. Atherosclerosis **318**: 76-82

Zhang Q, Zhou J, Zhang B, Jia W, Wu E (2020) Automatic epicardial fat segmentation and quantification of CT scans using dual U-Nets with a morphological processing layer. IEEE Access **8**: 128032-128041

Zlokolica V, Krstanović L, Velicki L, Popović B, Janev M, Obradović R et al (2017) Semiautomatic epicardial fat segmentation based on fuzzy c-means clustering and geometric ellipse fitting. Journal of Healthcare Engineering **2017**: 5817970
